# Genotype V Japanese Encephalitis Virus Is Emerging

**DOI:** 10.1371/journal.pntd.0001231

**Published:** 2011-07-05

**Authors:** Ming-Hua Li, Shi-Hong Fu, Wei-Xin Chen, Huan-Yu Wang, Yu-Hong Guo, Qi-Yong Liu, Yi-Xing Li, Hui-Ming Luo, Wa Da, Dun Zhu Duo Ji, Xiu-Min Ye, Guo-Dong Liang

**Affiliations:** 1 State Key Laboratory for Infectious Disease Prevention and Control, Institute for Viral Disease Control and Prevention, Chinese Center for Disease Control and Prevention, Beijing, China; 2 National Institute for Communicable Disease Control and Prevention, Chinese Center for Disease Control and Prevention, Beijing, China; 3 Chinese Center for Disease Control and Prevention, Beijing, China; 4 Tibet Center for Disease Control and Prevention, Tibet, China; Centre for Cellular and Molecular Biology (CCMB), India

## Abstract

Japanese encephalitis (JE) is a global public health issue that has spread widely to more than 20 countries in Asia and has extended its geographic range to the south Pacific region including Australia. JE has become the most important cause of viral encephalitis in the world. Japanese encephalitis viruses (JEV) are divided into five genotypes, based on the nucleotide sequence of the envelope (E) gene. The Muar strain, isolated from patient in Malaya in 1952, is the sole example of genotype V JEV. Here, the XZ0934 strain of JEV was isolated from *Culex tritaeniorhynchus*, collected in China. The complete nucleotide and amino acid sequence of XZ0934 strain have been determined. The nucleotide divergence ranged from 20.3% to 21.4% and amino acid divergence ranged from 8.4% to 10.0% when compared with the 62 known JEV isolates that belong to genotype I–IV. It reveals low similarity between XZ0934 and genotype I–IV JEVs. Phylogenetic analysis using both complete genome and structural gene nucleotide sequences demonstrates that XZ0934 belongs to genotype V. This, in turn, suggests that genotype V JEV is emerging in JEV endemic areas. Thus, increased surveillance and diagnosis of viral encephalitis caused by genotype V JEV is an issue of great concern to nations in which JEV is endemic.

## Author Summary

Japanese encephalitis virus (JEV) is a mosquito-borne virus that causes Japanese encephalitis (JE) with significant morbidity and mortality. Five genotypes (genotype I–V) have been identified based on the nucleotide sequence of viral envelope (E) gene of JEV. To date, the only known strain of genotype V is Muar strain, isolated from patient in Malaya in 1952. Since then, no genotype V JEV has been detected in the world. In this study, the JEV strain, XZ0934, was isolated from mosquito samples collected in China in 2009. The full-length genome sequences of the XZ0934 strain was determined and founded to be the second strain of genotype V JEV based on the phylogenetic analysis using the complete genome and structural gene sequences. This suggests that genotype V JEV is re-emerging after 57 years (1952–2009). Therefore, increased surveillance and more effective diagnosis for cases of JE caused by genotype V JEV are needed.

## Introduction

Japanese encephalitis (JE), which is caused by JE virus (JEV), is one of the most important viral encephalitis in the world [1]–[4]. It is prevalent mostly in Asia including eastern Asia [5]–[7], southern Asia [8] and southeast Asia [9], [10]. JE has extended its geographic range to the south Pacific region, including Australia [11], [12]. An estimated 3 billion persons live in countries where JE is endemic [1]–[3].

JEV has a zoonotic transmission cycle between mosquitoes (principally of the genus *Culex*) and vertebrate hosts such as bats, water birds and pigs [1], [4]. Human beings contract JEV when bitten by infected mosquitoes. Around 35,000–50,000 JE cases are reported each year, of which 10,000–15,000 are fatal [1]–[3], [13]. Approximately 50% of JE patients present severe neurological and mental sequelae such as motor deficits, and cognitive and language impairment [14]–[16].

JEV is a member of the genus *Flavivirus*, family *Flaviviridae* [1]. Like other flaviviruses, the JEV genome is a single-stranded positive-sense RNA of approximately 11 kb in length. It is capped at its 5′ end and has a single open reading frame (ORF) that encodes a polyprotein. The ORF is flanked by 5′ and 3′ untranslated regions (UTRs). The viral structural proteins are encoded by the 5′ one-third of the ORF and consist of the capsid (C), membrane (M; formed by proteolytic cleavage of its precursor protein PrM) and envelope (E) proteins. The remaining 3′ region encodes non-structural proteins (NS1 to NS5) [1], [17].

JEVs have been divided into five genotypes (genotype I, II, III, IV, V), based on nucleotide sequence of E gene [18]. Genotypes I-IV have been isolated from many vectors [19]–[21], bats [22], and patients [7], [8], [21], [23], [24] in Asia (including eastern, southern and southeast Asia) and Australia. To date, the Muar strain, which was isolated from specimens of brain tissues of patients with viral encephalitis in Malaya in 1952, is the only example of genotype V JEV [18], [25]. Since that time, no genotype V JEV has been detected. In this study, genotype V JEV was isolated from *Culex tritaeniorhynchus* collected in China in 2009. This suggests that genotype V JEV is re-emerging in Asian country after a 57 year hiatus.

## Materials and Methods

### Cell cultures

C6/36 (*Aedes albopictus*) cell line was grown in minimal essential medium (HyClone) with Hanks' salt solution supplemented with 10% fetal bovine serum (FBS, HyClone), 2 mM glutamine, 0.12% NaHCO_3_, and 100 U ml^−1^ penicillin and streptomycin. Cells were propagated and maintained at 28°C [21]. BHK-21 cells were grown in minimal essential medium (HyClone) with Earl's balanced salt solution supplemented with 10% FBS, 2 mM glutamine, 0.12% NaHCO_3_, and 100 U ml^−1^ penicillin and streptomycin. BHK-21 cells were propagated and maintained at 37°C under a 5% CO_2_ atmosphere [21].

### Mosquito collection

An arbovirus survey was conducted in Tibet in the summer of 2009. Mosquitoes were collected in Mainling County (altitude 2900 m) and Medog County (altitude 1000 m) in the Nyingchi area of Tibet. Mosquito samples were collected using mosquito-trapping lamps (Wuhan Lucky Star Environmental Protection Tech Co., Ltd., Hubei, China) in the evening. Collection locations were proximal to sites of frequent human activity. Collection nets containing mosquitoes were frozen for 30 min at −20°C and transferred onto an ice plate for determination of mosquito species (blood-fed and male mosquitoes were discarded). Female mosquitoes were identified to species level by morphologic characteristics and sorted into pools of 100 specimens according to species. The pools were put into collection tubes individually and stored in liquid nitrogen [26], [27].

### Virus isolation

Mosquito pools were added to 1.5 ml minimal essential medium (HyClone), supplemented with 2 mM glutamine, 0.12% NaHCO_3_, and 100 U ml^−1^ penicillin and streptomycin, followed by grinding in a pre-cooled sterile plastic grinding tube using a TissueLyser (GIAGEN, Germany). Homogenized samples were centrifuged at 17,000× g in a microcentrifuge for 20 min at 4°C, and the clarified supernatants were used to inoculate monolayers of BHK-21 and C6/36 cells and incubated at 37°C and 28°C, respectively. The cells were observed daily to check for development of cytopathic effects (CPE). A sample was regarded as virus-positive if it caused CPE in successive cell passages [26], [27].

### Virus purification

Viral supernatants were applied to six-well plates (Corning, USA) of confluent BHK-21 cells and incubated for one hour. Plates were first overlaid with medium containing 75% agarose and then with medium containing neutral red vital stain after three days incubation at 37°C in a 5% CO_2_ incubator. Plaques of different sizes and shape were shattered in 500 ul MEM medium after being picked out using a sterile pipette tip. As described previously [28], this process was repeated until a single plaque-shaped virus was obtained.

### RNA extraction, reverse transcription and PCR amplification

Viral RNA was extracted from 140 ul supernatant from virus-infected BHK-21 cell cultures using a Viral RNA Mini Kit (QIAGEN, Germany) according to the manufacturer's instructions. cDNA was synthesized using a Ready-to-Go You-Prime First-Strand Beads Kit (GE healthcare, UK) and random hexanucleotide primers. PCR amplification using universal primers specific for flaviviruses, alphaviruses and bunyaviruses was conducted for identification of virus isolates [29]. Primers (Table 1) were designed for full-length genome amplification and sequencing of JEV using the PREMIER Primer 5 software package. Thermal cycling parameters were as follows: one cycle of denaturation (94°C, 5 min) followed by 35 cycles of 94°C denaturation for 30 s, 55°C annealing for 30 s, and 72°C extension for 1 min. The programme ended with an extension step at 72°C for 10 min. Amplified products were examined by agarose gel electrophoresis (1%), purified using a QIAquick Gel Extraction kit (QIAGEN, Germany), and then sequenced directly. Sequencing of the 5′ UTR and 3′ UTR were determined by using 5′ RACE and 3′ RACE system for Rapid Amplification of cDNA Ends (Invitrogen) respectively. 5′ RACE was performed according to standard protocols (Invitrogen 5′ RACE kit). 3′ RACE was performed by first adding a polyA tail using PolyA polymerase (New England Biolabs) and then conduct RT-PCR with gene specific primers and an oligo-dT-adapter primer.

**Table 1:** Primers used for RT-PCR and sequencing of XZ0934 in this study.

Primer	Sequence (5′–3′)	Position	Orientation
JEV-V-1	AGAAGTTTATCTGTGTGAACT	1–21	Sense
JEV-V-2	CACAAGAGCATAGCCTGGAT	868–887	Antisense
JEV-V-3	AGGGACTAATAGATGTTGGG	563–582	Sense
JEV-V-4	TCAGAGTGATGGAAGGAG	1495–1512	Antisense
JEV-V-5	GCGAACGACAAACCAACA	1080–1097	Sense
JEV-V-6	GACGTAATGCCAAACCCA	2951–2968	Antisense
JEV-V-7	GCCATTGACATAACCAGAA	2490–2508	Sense
JEV-V-8	ATCAAGGCCAAATGAACT	3755–3772	Antisense
JEV-V-9	ATACGGCCCATGAAGCAT	3480–3497	Sense
JEV-V-10	CTCGAGCCATTATCCTGTA	4674–4692	Antisense
JEV-V-11	GGCCATCATCCCAGCAGCT	4553–4571	Sense
JEV-V-12	CCTCTAAGAGCTTCTGCCAT	5301–5320	Antisense
JEV-V-13	AACAGCTGTTCTAGCGCC	5261–5278	Sense
JEV-V-14	CCATTGTGAAAGCCTTCTCC	6149–6168	Antisense
JEV-V-15	AGAGTAGGCAGAAATCCGAA	5988–6007	Sense
JEV-V-16	GAGGCTAGTGATGTTGTCAC	7107–7126	Antisense
JEV-V-17	GATGCAACGAAAGGGCATAG	6677–6696	Sense
JEV-V-18	TGAGACAAACCCTTTCTCCA	7876–7895	Antisense
JEV-V-19	CCTGTGGGATAATGGAGC	7538–7555	Sense
JEV-V-20	TTGGTGGTTTCGTCCAGCAC	8802–8821	Antisense
JEV-V-21	AATGTACTGGGTTAGTGG	8333–8350	Sense
JEV-V-22	CTTACGTCGGCCTGACTCCC	10465–10484	Antisense
JEV-V-23	CAAATGTGGCTCCTCCTCT	9972–9990	Sense
JEV-V-24	AGATCCTGTGTTCTTCCTCA	10964–10983	Antisense

### Multiple alignments and sequence analyses

The full-length genome of the XZ0934 strain was compiled using SeqMan in the Lasergene software package (DNASTAR). Nucleotide and amino acid sequence alignments were generated by ClustalX version 2.0.9 [22], [30]. Analysis of nucleotide and deduced amino acid sequence identities was performed using GeneDoc and MegAlign in the Lasergene software package (DNASTAR).

### Phylogenetic analysis

Full-length nucleotide sequences of 32 selected JEV strains of varying genotype isolated from different locations and sources, and across a number of years, were downloaded from GenBank (Table 2). The reported structural gene sequence of genotype V JEV (Muar strain) [25] was used to build phylogenetic trees. Neighbor-joining phylogenetic trees based on nucleotide sequences were constructed using MEGA version 4.0.2 [18], [31]. The robustness of phylogenetic constructions was evaluated by bootstrapping using 1000 replicates.

**Table 2:** Background information of 32 selected strains of Japanese encephalitis virus used in this study.

No.	Genotype	Strain	Year	Location	Source	GenBank accession no.
1	I	K94P05	1994	Korea	Mosquito	AF045551
2	I	KV1899	1999	Korea	Pig	AY316157
3	I	Ishikawa	1998	Japan	Mosquito	AB051292
4	I	JEV/sw/Mie/41/2002	2002	Japan	Pig	AB241119
5	I	JEV/sw/Mie/40/2004	2004	Japan	Pig	AB241118
6	I	SC04-17	2004	China	Mosquito	GU187972
7	I	HEN0701	2007	China	Pig	FJ495189
8	I	XJ69	2007	China	Mosquito	EU880214
9	I	XJP613	2007	China	Mosquito	EU693899
10	I	SH17M-07	2007	China	Mosquito	EU429297
11	I	JX61	2008	China	Pig	GU556217
12	II	FU	1995	Australia	Human	AF217620
13	III	Vellore P20778	1958	India	Human	AF080251
14	III	GP78	1978	India	Human	AF075723
15	III	014178	2001	India	Human	EF623987
16	III	04940-4	2002	India	Mosquito	EF623989
17	III	057434	2005	India	Human	EF623988
18	III	Nakayama	1935	Japan	Human	EF571853
19	III	JaGAr01	1959	Japan	Mosquito	AF069076
20	III	JaOH0566	1966	Japan	Human	AY508813
21	III	JaOArS982	1982	Japan	Mosquito	M18370
22	III	K87P39	1987	Korea	Mosquito	AY585242
23	III	p3	1949	China	Human	U47032
24	III	Beijing-1	1949	China	Human	L48961
25	III	SA14-14-2	1954	China	Vaccine strain	AF315119
26	III	HW	1988	China	Pig	AY849939
27	III	WHe	1988	China	Pig	EF107523
28	III	SH0601	2006	China	Pig	EF543861
29	III	Ling	1965	Taiwan	Human	L78128
30	III	CH1392	1990	Taiwan	Mosquito	AF254452
31	III	T1P1	1997	Taiwan	Mosquito	AF254453
32	IV	JKT6468	1981	Indonesia	Mosquito	AY184212

To better understand the phylogenetic relationship between genotype V JEV and other flaviviruses, full-length nucleotide sequences of previously published JEV strains and other flaviviruses were downloaded from GenBank, including sequences from Murray Valley encephalitis virus (MVEV), West Nile virus (WNV), Kunjin virus (KUNV), St. Louis encephalitis virus (SLEV), Dengue virus 1 (DENV1, Dengue virus 2 (DENV2), Dengue virus 3 (DENV3), Dengue virus 4 (DENV4), Yellow fever virus (YFV), Powassan virus (POWV), Langat virus (LANV), Louping ill virus (LIV), Tick-borne encephalitis virus (TBEV) and Culex flavivirus (Table 3).

**Table 3:** Background information of 14 selected strains of Flaviviruses used in this study.

Virus	Strain	GenBank accession no.
Murray Valley encephalitis virus (MVEV)	MVE-1-51	NC_000943
West Nile virus (WNV)	ArB3573/82	DQ318020
Kunjin virus (KUNV)	MRM61C	D00246
St. Louis encephalitis virus (SLEV)	Kern217	DQ525916
Dengue virus 1 (DENV1)	SG(EHI)D1227Y03	FJ469909
Dengue virus 2 (DENV2)	D2/SG/05K4155DK1/2005	EU081180
Dengue virus 3 (DENV3)	D3/H/IMTSSA-MART/1999/1243	AY099337
Dengue virus 4 (DENV4)	341750	GU289913
Yellow fever virus (YFV)	17D/Tiantan	FJ654700
Powassan virus (POWV)	Spassk-9	EU770575
Langat virus (LANV)	TP21	NC_003690
Louping ill virus (LIV)	369/T2	NC_001809
Tick-borne encephalitis virus (TBEV)	Toro-2003	DQ401140
Culex flavivirus	Tokyo	AB262759

## Results

### Virus isolation and purification

After homogenized supernatants were inoculated onto monolayers of BHK-21 and C6/36 cells, a single pool containing 100 specimens of *Culex tritaeniorhynchus* yielded a virus isolate designated XZ0934. The supernatant of pool XZ0934 caused cytopathic effects (CPE) in BHK-21 and C6/36 cells in successive cell passages. The C6/36 cells became aggregate, and showed fusion and shedding while the BHK-21 cells became aggregate and began shedding by 72 h post-infection. All plaques in BHK-21 cell monolayers were of identical size (mean 1.5 mm, n = 10). Two plaques were picked from them and subjected to a second round of plaque purification. The resultant data were consistent with the former.

### Virus identification

Viral RNA was extracted and amplified by PCR using primers specific for flaviviruses, alphaviruses and bunyaviruses. XZ0934 was positive when primers specific for flaviviruses (FU1/cFD2) [29] were used, and nucleotide sequencing confirmed that XZ0934 was a JEV. To ensure the consistency of different viral plaques, six purified plaques were picked and amplified using flavivirus-specific primers (FU1/cFD2). The nucleotide and amino acid sequence identities of the six purified plaques were 100%. This indicates that each of the six purified plaques was generated by an identical JEV strain. Therefore, one plaque was selected for full-length genome amplification and sequencing.

### Determination of viral genome sequence

Recent reports have suggested that JEVs currently circulating in China belong to genotypes I and III [5]–[7], [21]–[23]. Thus, 32 primers were designed using the complete sequences of genotype I JEV Ishikawa (GenBank accession number AB051292) and 48 from the sequence of genotype III JEV Beijing-1 (GenBank accession number L48961). These were used for amplification of the entire XZ0934 genome. PCRs were positive with 4 genotype I and 10 genotype III primers. Based on obtained nucleotide sequences, primers were designed to close the majority of gaps between assembled contigs by PCR amplification in order to determine the whole genome of XZ0934. A further 24 primers (Table 1) were designed and used to verify the accuracy of sequencing. The complete genome (10,983 nt) of XZ0934 was sequenced (GenBank accession no. JF915894) and found to possess one open reading frame (ORF). When the complete genome sequence of isolate XZ0934 was compared with those of 62 known JEV isolates (genotypes I–IV) in Genbank, sequence identities varied from 78.6% (KV1899, K94P05) to 79.7% (CC27-L1) and amino acid sequence identity from 90.0% (KV1899, K94P05) to 91.6% (K87P39). Thus, these data reveal low similarity between XZ0934 and genotype I–IV JEVs.

Because the structural gene sequence of genotype V (Muar) has been reported [25], an identity analysis of JEV structural genes (C, PrM, M, E) of XZ0934, Muar and other selected genotype I–IV JEV strains was conducted (Table 4). C gene sequence homology varied from 78.2% (G IV, JKT 6468) to 88.5% (G V, Muar) for nucleotides and 72.4% (G IV, JKT 6468) to 85.8% (G V, Muar) for amino acids. That of the PrM gene varied from 71.7% (G IV, JKT 6468) to 84.1% (G V, Muar) for nucleotides and 81.5% (G IV, JKT 6468) to 90.2% (G V, Muar) for amino acids. M gene sequence homology varied from 80.0% (G IV, JKT 6468) to 95.6% (G V, Muar) for nucleotides and 85.3% (G IV, JKT 6468) to 100.0% (G V, Muar) for amino acids. E gene sequence homology varied from 77.0% (G I, Ishikawa) to 86.0% (G V, Muar) for nucleotides and 89.4% (G I, Ishikawa) to 93.2% (G V, Muar) for amino acids. These data demonstrate that the structural gene sequence homology of XZ0934 was higher with genotype V JEV (Muar) than with other genotype I–IV JEV strains.

**Table 4:** Sequence homology between XZ0934 and five genotype JEV strains in structural gene.

Genotype	Strain	Percentage homology of nucleotides (amino acids)			
		C	PrM	M	E
G I	Ishikawa (AB051292)	79.5% (77.2%)	78.3% (87.0%)	84.0% (93.3%)	77.0% (89.4%)
G II	FU (AF217620)	81.4% (78.7%)	74.3% (84.8%)	81.8% (94.7%)	77.5% (90.6%)
G III	p3 (U47032)	81.4% (78.0%)	76.8% (85.9%)	83.6% (93.3%)	77.4% (90.2%)
G IV	JKT 6468 (AY184212)	78.2% (72.4%)	71.7% (81.5%)	80.0% (85.3%)	77.5% (90.6%)
G V	Muar (Hasegawa et al.(25))	88.5% (85.8%)	84.1% (90.2%)	95.6% (100.0%)	86.0% (93.2%)

### Phylogenetic analysis

**Figure 1:**
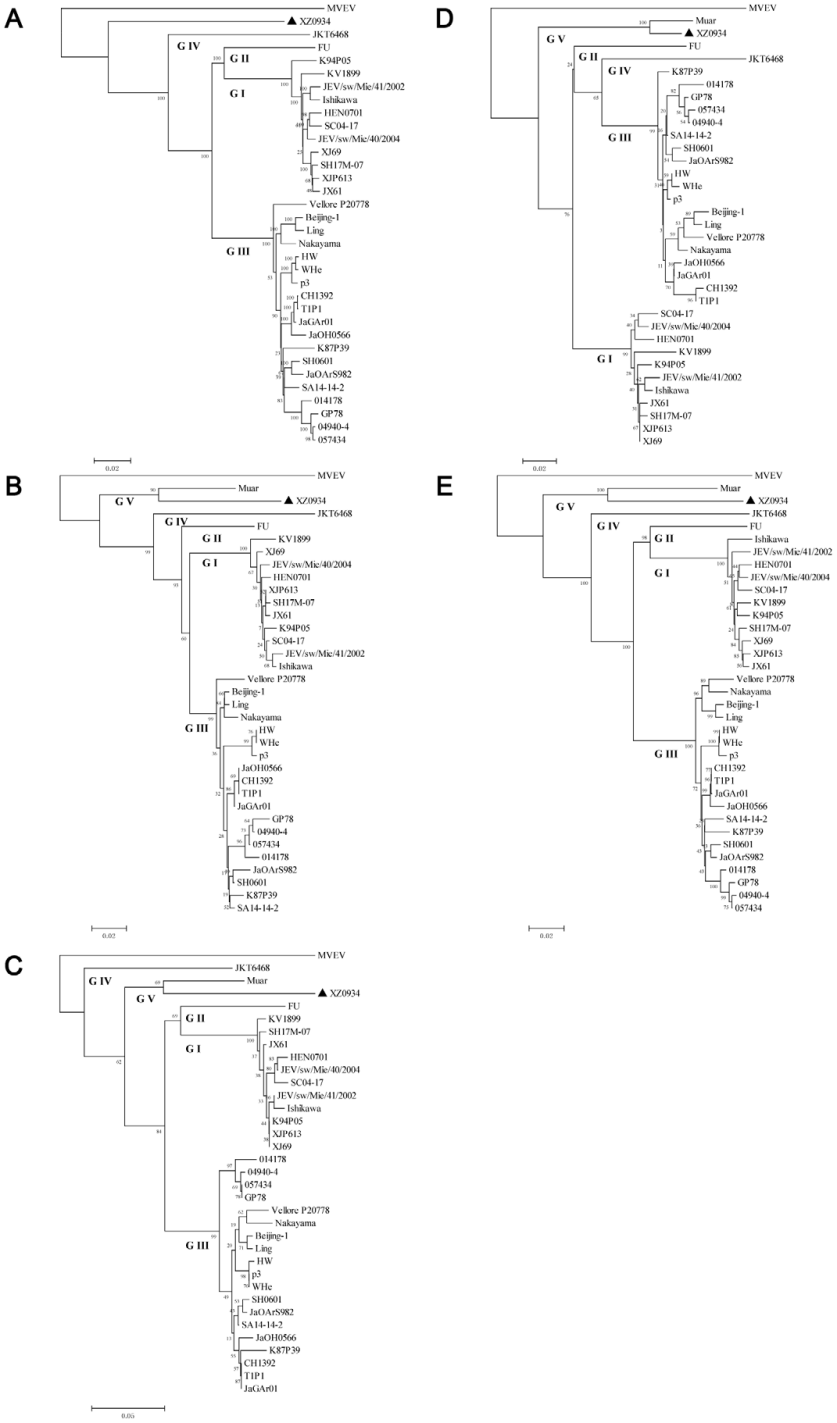
Phylogenetic analysis of XZ0934 and other JEV strains based on the nucleotide sequences. A) complete genome; B) C gene; C) PrM gene; D) M gene; E) E gene. Phylogenetic analyses were performed by the neighbor-joining method using MEGA version 4.0.2 software package (www.megasoftware.net). The tree was rooted using Murray Valley encephalitis virus (MVEV) strain MVE-1-51 as an outgroup. Bootstrap probabilities of each node were calculated using 1000 replicates. Scale bars indicate the number of nucleotide substitutions per site.

To establish the phylogenetic relationship between XZ0934 and other JEV strains, a phylogenetic tree was constructed using the complete genome sequences of XZ0934 and 32 selected JEV strains (genotypes I–IV). Murray Valley encephalitis virus (MVEV) was used as an outgroup. Five distinct phylogenetic groups were identified. The XZ0934 strain, which was isolated from China, formed a branch divergent from other genotype I–IV JEV strains (Figure 1A). Therefore, XZ0934 should be regarded as a novel, non-genotype I–IV, JEV isolate.

To study their phylogenetic relationship, a phylogenetic tree was constructed using the reported structural gene nucleotide sequences of Muar [25], XZ0934, and other JEV strains (genotype I–IV). No matter which structural gene was used to construct the phylogenetic tree, the topology was similar. Five distinct phylogenetic groups were evident in each tree. XZ0934 and Muar fell into the same group when the tree was constructed using the C (Figure 1B), PrM (Figure 1C), M (Figure 1D) or E (Figure 1E) genes. This result suggested that XZ0934 was a novel genotype V JEV isolate.

**Figure 2:**
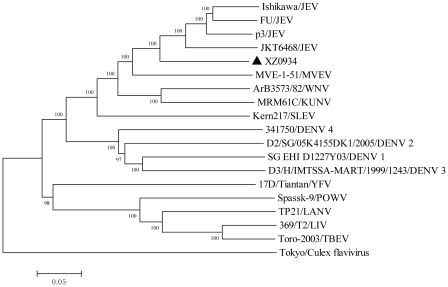
Phylogenetic analysis of XZ0934 and other flaviviruses constructed using complete genome sequences. Phylogenetic analyses were performed by the neighbor-joining method using MEGA version 4.0.2 software package (www.megasoftware.net). Bootstrap probabilities of each node were calculated using 1000 replicates. Scale bars indicate the number of nucleotide substitutions per site.

A phylogenetic tree was also constructed using genomic nucleotide sequences in order to understand the phylogenetic relationship between XZ0934 and other flaviviruses. Data indicated that XZ0934 was indeed a JEV rather than any of the other 14 flaviviruses (Figure 2).

## Discussion

In recent years, the sequence of the JEV viral envelope (E) gene has been used by various authors to perform phylogenetic analyses [18], [21], [32], [33]. Based on the resultant data, JEV strains have been divided into five genotypes (genotypes I-V) [18]. Genotypes I and III are distributed widely in Asia, including Japan, Korea, China, India, Vietnam and Philippines. Genotype II includes isolates from southern Thailand, Malaysia, Indonesia, and northern Australia. Genotype IV has been isolated only in Indonesia [18]. The Muar strain, isolated in Malaya in 1952, is regarded as the only genotype V JEV isolate [18], [25], [33]. In this study, phylogenetic analysis of structural genes and whole genome sequences also suggested the existence of five JEV genotypes. Thus, the E gene is confirmed to be a useful phylogenetic marker for JEV.

Primers designed for JEV genotypes I and III were used for full-length amplification of XZ0934. Of these, only a few (4/32 genotype I and 10/48 genotype III) resulted in successful amplification. This suggests a low whole genome sequence homology between XZ0934 and genotype I and I JEV isolates. In order to further understand the differences between XZ0934 and other JEV strains (genotype I–IV), an identity analysis was conducted using the full-length nucleotide sequences of XZ0934 and 62 known JEV isolates (genotypes I–IV) in Genbank. Data suggested that XZ0934 and the genotype I–IV JEV strains were dissimilar. The nucleotide sequence identity varied from 78.6% to 79.7% and amino acid sequence identity from 90.0% to 91.6%. Indeed, the sequence divergence ranged from 20.3% to 21.4% (nt) and 8.4%–10.0% (aa). It has been suggested that the nucleotide sequence divergence between different JEV genotypes is ∼10% [13]. The sequence divergence (20.3%–21.4%) between XZ0934 and the genotype I–IV JEVs was greater than 10%, suggesting that XZ0934 is not a member of JEV genotypes I–IV. To confirm that XZ0934 was a JEV and not some other flavivirus, 14 flavivirus strains, including mosquito-borne and tick-borne flaviviruses, were used to build a phylogenetic tree. The data indicated that XZ0934 was indeed a JEV (Fig. 2).

Four viral encephalitis cases were reported in Malaya (n = 1) and Singapore (n = 3) in the summer of 1952. All patients exhibited high fever, vomiting, headache, disturbance of consciousness, stiff neck and deep coma with rapid progression to death by respiratory failure. Four virus strains were isolated from brain tissue specimens and identified as JEV by neutralization test using the Japanese Nakayama JEV strain [24]. Of these, the Muar strain, isolated from a 19-year-old male patient in Malaya in 1952, has been assigned to genotype V based on the E gene sequence [18], [25], [32], [33]. During the following 57 years (1952–2009), no genotype V JEV has been reported. In this study, XZ0934, isolated from *Culex tritaeniorhynchus* collected in China, has been identified as a genotype V JEV, based on phylogenetic analysis using both full-length genome and structural gene nucleotide sequences. This represents only the second instance of isolation of genotype V JEV worldwide since 1952. Thus, genotype V JEV is not limited to southeast Asia and has begun to be emerge in the world.

Many factors may contribute to spread of JEV [1], [2], such as changed agricultural practices (which provide new breeding sites for mosquitoes), animal husbandry (which provides host animals for transmission) [34], migrating birds and even wind-blown mosquitoes [11], [35]. Each of the five known JEV genotypes originated in the Indonesia-Malaysia region [18], so why has genotype V JEV not been detected for 57 years? How did it spread to China from southeast Asia, a distance of thousands of kilometers? Does this virus exist somewhere along the path from Malaysia to China? All these issues are worthy of further study. Moreover, genotype V JEV was first isolated from human specimens, suggesting a high pathogenicity and the possibility of viral encephalitis. Therefore, increased surveillance and more effective diagnosis of viral encephalitis caused by genotype V JEV is an issue of great concern to nations in which JEV is endemic.
